# Higher Testing Coverage Associated With a Lower COVID-19 Mortality Rate: Insights From Italian Regions

**DOI:** 10.1017/dmp.2020.236

**Published:** 2020-07-14

**Authors:** Andrea Saglietto, Giovenale Moirano, Matteo Anselmino, Gaetano Maria De Ferrari

**Affiliations:** 1 Division of Cardiology, Department of Medical Sciences, Città della Salute e della Scienza Hospital, University of Turin, Italy; 2 Epidemiology Unit, Department of Medical Sciences, Città della Salute e della Scienza Hospital, University of Turin, Italy

**Keywords:** COVID-19, epidemiology, Italy

## Abstract

Different coronavirus disease (COVID-19) testing approaches have been implemented among Italian regions, reflected in heterogeneous testing rates. We analyzed the number of COVID-19-related deaths in relation to the number of tests performed among the most affected Italian regions. We showed that regions with the highest number of tests performed (Veneto and Toscana) had the lowest 30-day crude mortality rate per 100 000 inhabitants. In addition, an inverse association between crude mortality rates and tests performed (mortality rate ratio for a unit increase in tests per 1000 inhabitants: 0.92; 95% CI: 0.89–0.94) was observed. Early identification and isolation of active cases (including asymptomatic or mildly symptomatic subjects) could have had an important effect in lowering COVID-19 mortality.

The first locally transmitted case of severe acute respiratory syndrome coronavirus 2 (SARS-CoV-2) in Italy was detected on February 21, 2020. The increasing number of cases forced the Italian Government to introduce specific measures to restrict social contact at the beginning of March.^1,2^ These measures were further tightened on March 22, 2020, when the Italian Government declared a total lockdown, only allowing essential activities.^3^ As of May 6, 2020, Italy was the most affected European country in terms of coronavirus disease (COVID-19)-related deaths, with more than 29 000 fatal events recorded. The crude COVID-19 case-fatality ratio (CFR) in Italy was one of the highest in the world (~14%).^4^ One of the reasons might be that initial Italian testing coverage has been lower than other European countries^5^; a low coverage of the population at risk implies a lower chance to find positive asymptomatic individuals, thus underestimating the total number of cases, yielding an increased CFR. However, given that governance of the Italian Health System is decentralized, different COVID-19 testing approaches have been adopted by Italian regions, as reflected in the extremely variable testing rates.^6^

## RATIONALE

Testing subjects with diagnostic tests could facilitate early identification of active cases (including asymptomatic or mildly symptomatic subjects) and could have an effect in lowering COVID-19 mortality by isolating cases that would otherwise continue to spread the virus in the susceptible and vulnerable population. Heterogeneity in testing approaches within the same country offers the opportunity to study whether different testing strategies had an effect in lowering the COVID-19 mortality, since heterogeneity in other potential confounders (quality and timing of restrictive measures, or population age-structure) is limited within the same country.^7^

## METHODS

Since the number of confirmed cases is affected by testing coverage, we focused on the number of COVID-19-related deaths, which are less likely to be affected by testing approaches, in the 7 most affected Italian regions as of May 6, 2020. First, the cumulative mortality rate curve per 100 000 inhabitants was plotted for each region, starting from the day with at least 5 cumulative COVID-19 deaths (considered as *Day 1*) up to 30 days (considered as *Day 30*). We then computed crude mortality rates (CMRs) at Day 30 with a corresponding 95% confidence interval (exact Poisson method). Finally, we fitted a multilevel Poisson regression to the cumulative number of COVID-19-related deaths that occurred in the 30 days, using the population as offset and introducing a region-specific random effect. The model was applied to study the mortality rates in relation to the cumulative number of tests performed in each region over the 30-day observation period. Tests performed were expressed as both the number of tests per identified case and the number of tests per 1000 inhabitants. Regression analysis was adjusted for the proportion of inhabitants over 65 years of age. All data were obtained from the Italian Civil Protection website,^6^ and an analysis was performed with the R Foundation (R Project for Statistical Computing, Vienna, Austria).

## RESULTS

**FIGURE 1:**
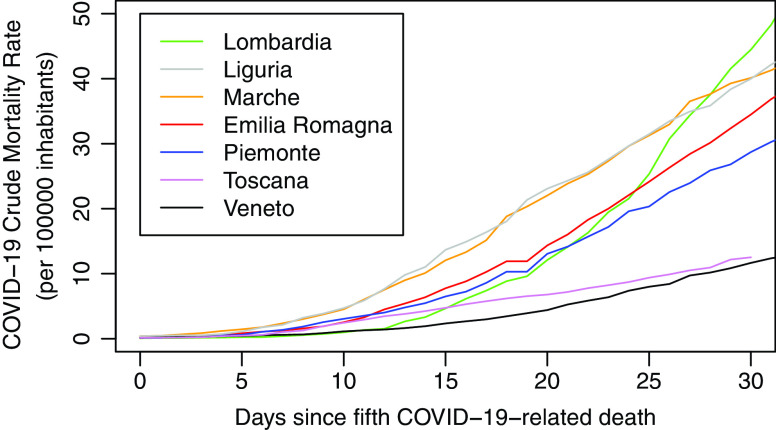
Cumulative Mortality Rate Curves for Different Italian Regions.

Figure 1 reports cumulative mortality rate curves for the 7 Italian regions. Veneto and Toscana, by far, experienced lower mortality rates compared with other regions (Table 1). Interestingly, Veneto and Toscana are the 2 regions with the highest number of tests performed (see Table 1). Considering regions altogether, we observed an inverse association between CMRs and tests performed (mortality rate ratio [MRR] for a unit increase in tests per identified case: 0.87, 95% CI: 0.84–0.90; MRR for a unit increase in tests per 1000 inhabitants: 0.92; 95% CI: 0.89–0.94).

**TABLE 1:** COVID-19 Incidence Rates, COVID-19 Mortality Rates, and Tests Performed in Different Italian Regions in the 30 Days After the Fifth COVID-19-Related Deaths

Region	First Day with 5 Cumulative Deaths	Crude Incidence Rates at Day 30 (per 100 000 inhabitants)	Crude Mortality Rate at Day 30 (per 100 000 inhabitants)	Mortality Rate Ratio at Day 30 (95% CI)	Tests Per Identified Case at Day 30	Tests Per 1000 Inhabitants at Day 30
Veneto	04 Mar	206.1	10.8	Ref	11.9	24.5
Toscana	12 Mar	180.4	12.2	1.12 (0.99–1.27)	10.7	19.3
Piemonte	07 Mar	283.8	26.8	2.47 (2.23–2.74)	3.1	8.8
Emilia Romagna	01 Mar	315.6	32.3	2.98 (2.70–3.30)	3.8	11.4
Liguria	08 Mar	293.4	38.4	3.54 (3.22–4.08)	3.7	10.0
Marche	07 Mar	292.7	39.3	3.62 (3.22–4.08)	3.4	10.2
Lombardia	24 Feb	305.2	41.5	3.83 (3.50–4.20)	2.5	7.6

## DISCUSSION

Our study shows that Italian regions experienced different COVID-19 mortality rates. This may be only partially explained by differences in the population age-structure (the proportion of those over 65 years old being 22.9% in Veneto and 22.5% in Lombardia, as an example) or the COVID-19-related deaths reported. COVID-19 mortality rates, instead, were associated with different testing approaches adopted by the investigated Italian regions. In particular, our results suggest that higher testing rates were associated with lower CMRs. Early identification of active cases (including asymptomatic or mildly symptomatic subjects) might have had an effect in lowering COVID-19 mortality, by isolating cases that would otherwise continue to spread the virus in the susceptible population and possibly preventing overwhelming of local health care systems. Increasing testing coverage and active tracing of contacts may play a relevant role in limiting the public health impact of the COVID-19 outbreak, by significantly reducing deaths in the population.

### Limitations

The present results must be interpreted with caution, given that the study is based on aggregated regional data. As most of ecological studies, systematic differences between regions in exposure (tests performed) and outcome (COVID-19-related deaths) measurements are possible. However, since this study was performed within the same country, strong heterogeneity in these measurements is unlikely. In addition, comparing data between regions from the same country should have limited the heterogeneity in other potential confounders (eg, timing and quality of restrictive measures).

## CONCLUSION

The present analysis on aggregate regional Italian data suggests that higher COVID-19 testing rates were associated with lower mortality during the first month of the outbreak. These results prompt urgent studies investigating the impact that preventive strategies, including the extent of testing coverage, may exert in terms of reducing COVID-19 mortality, both in high- and low-transmission settings.

## References

[1] Gazzetta Ufficiale. Il presidente del consiglio dei ministri. March 9, 2020. https://www.gazzettaufficiale.it/eli/id/2020/03/09/20A01558/sg. Accessed May 6, 2020.

[2] Saglietto A , D’Ascenzo F , Zoccai GB , De Ferrari GM. COVID-19 in Europe: the Italian lesson. Lancet. 2020;395(10230):1110‐1111. 32220279 10.1016/S0140-6736(20)30690-5 PMC7118630

[3] Gazzetta Ufficiale. Il presidente del consiglio dei ministri. March 22, 2020. https://www.gazzettaufficiale.it/eli/id/2020/03/22/20A01807/sg. Accessed May 6, 2020.

[4] Center for Systems Science and Engineering, Johns Hopkins University. COVID-19 dashboard. 2020. https://gisanddata.maps.arcgis.com/apps/opsdashboard/index.html#/ bda7594740fd40299423467b48e9ecf6. Accessed May 6, 2020.

[5] Our World in Data. Statistics and Research. Coronavirus (COVID-19) testing. 2020. https://ourworldindata.org/covid-testing. Accessed May 6, 2020.

[6] GitHub, Inc. COVID-19. 2020. https://github.com/pcm-dpc/COVID-19/tree/master/dati-regioni. Accessed May 6, 2020.

[7] Kashnitsky I , Schöley J. Regional population structures at a glance. Lancet. 2018;392(10143):209‐210. 30043748 10.1016/S0140-6736(18)31194-2

